# Monthly Botanical Report

**Published:** 1810-12

**Authors:** 


### MONTHLY BOTANICAL REPORT.

Our last Report being occupied by an account of the Hortus Kewensis, the periodical works on Botany were necessarily neglected. We shall now proceed to notice the four last published numbers of the Botanical Magazine. We shall enumerate all the plants in Mr. Ker's* department in succession, without interrupting the series by those of Dr. Sims.

Alöe *arborescens*, the tree aloe; one of the most gigantic of the species, but which was considered by Linnæus as a variety of *perfoliata*.

Alöe *arachnoides*, var. *reticulata*. This is one of the most diminutive species, nearly allied to the Cushion Aloe.

One can hardly conceive that these two plants can be properly united under one genus, differing so much as they do in habit, in foliage, and in the form, as well as colour of the flower. The genus ought, if not entirely separated, to be divided into sections.

Alöe *lingua*; the tongue-Aloe. All these three afford examples of as many different sections, if not of distinct genera.

Alöe *picta*. This would fall under the same section as *arborea*; as the next, Alöe *carinata*, would unite with *lingua*.

Alöe *depressa*. This species was considered by Linnæus as a variety of *perfoliata*, and would consequently be arranged under the first-mentioned section. To this plant an outline of a diminished figure of the whole plant is added. This is a most useful addition; without which the full-sized representation on so small a plate can hardly convey an intelligible idea of such very large plants. We can but wish that this method had been more generally adopted in cases where a small portion of a plant is insufficient to give a proper notion of the whole. We are happy to receive so many representations of succulent plants, which, hardly admitting of being preserved as dried specimens, are, on that account, more particularly desirable.

Hæmanthus *puniceus*. In a former number, Mr. Ker had observed, that Hæmanthus *multiflorus* probably did not properly belong to this genus; but he here acknowledges that it bears a red berry, which corresponds with the rest of the genus, and, in consequence, desires his former observation to be annulled.

Medeola *virginiana*. The roots are said to be eaten by the Indians, and to have a taste like cucumber.

Anthericum *alöoides*. Mr. Ker has elsewhere remarked, that this genus requires reforming and separating. The one here figured, *frutescens* and *longiscapum* of Jacquin, are all three closely allied, are natives of the Cape of Good Hope, and have yellow flowers and succulent leaves.

* This botanist being every where quoted by his present name in the Hortus Kewensis, we shall in future follow this example, and drop that of Gawler, though the letter G still continue to point out his articles.

Allium

Allium *cernuum.* There is a singularity in the form of germen in this species, which we do not recollect to have seen described in any other; it is triangular, and the angles are elongated at the top of the germen into a bifid process.

In a note added to this article, Mr. Ker remarks, that Allium *striatum* is not a native of the Cape, as he had before stated it to be on the authority of Jacquin, but of North America; and that Ornithogalum *bivalve*, of Linnæus, is the same plant.

Albuca *vittata* appears to be a species not before described.

Allium *flavum.* Yellow flowers are uncommon in this genus; this and *moly* are the only ones we recollect. Mr. Ker, in a former article, No. 1143, corrected a mistake that he had fallen into at No. 973, in giving a wrong plant for Allium *paniculatum.* He now directs, that the observation there made, that "the pedicles are intermixed with small round bulbs" should be expunged, as it belongs to *oleraceum*, between which and *paniculatum*, these bulbs are one of the chief distinctions.

In the above enumeration, we have brought all the aloes together, though intermixed with the other plants in the publication.

In Dr. Sims's department in the same four numbers, we find:

Phlox *pilosa* and *amœna*, two nearly allied species; the former is supposed to be the *aristata* of Michaux, and the latter his *philosa.* Both these plants were introduced by Mr. Fraser, of Sloane-square, who, it is here remarked, has made seven voyages to North America, for the laudable purpose of increasing our knowledge in the vegetable productions of that part of the world.

Claytonia *alsinoides.* This species, according to Dr. Sims, is distinct from *sibirica*, for which it has been generally taken. Introduced from Nootka Sound, by Mr. Archibald Menzies.

Goodlo *pubescens.* A decandrous papilionaceous plant, from Van Diemen's Island; which country being subject to a frost, it is probable, that its vegetables will be found sufficiently hardy to endure our winters without shelter.

Lupinus *Nootkatensis.* Another discovery of Mr. A. Menzies, on the north-west coast of America, and already become very common in our gardens.

Othonna *amplexicaulis.* From the singularity of its foliage, this plant makes a very picturesque drawing. It is a rare species, and was communicated by Mr. Knight, nurseryman, King's-road, Chelsea.

Billardiera *mutabilis.* An elegant little shrub from New South Wales.

Lonicera *flava.* Supposed to be a new species of woodbine, from North America, discovered by Mr. Fraser, of Sloane-square.

Lobelia *lutea*; from the Cape of Good Hope. Dr. Sims queries whether this properly belongs to the genus lobelia; to us the reversion of the flower does not seem at all sufficient for a separation; neither is this singular, we know at least of one other species, in which the same take place; and in this species, likewise, the tube is nearly, if not altogether, wanting.

*Mantisia*

*Mantisia saltatoria.* This is one of the most singular acitamineous plants we have seen. It is at the same time very beautiful. The airy-looking party-coloured *corollas*, have been fancifully compared to dancing girls. Dr. Sims thought it resembled the insect called mantis, whence his generic name. But adopting at the same time the former notion he has given it the specific name of *saltatoria:* and in English has called it opera girls. Though we were at first somewhat shocked at so whimsical and apparently unscientific a name, yet upon further consideration we do not see much to object to in it. Hitherto no attempt has been made to reduce the English names to a scientific form, and whilst ladies-tresses, friars-cowl, Jupiters distaff, love-lies-bleeding, fresh-water-soldier, fair-maids of France, are to be found in the most scientific catalogue that this country has produced, we need not be over fastidious. We might perhaps go farther, and maintain that as names taken from a fancied similarity when converted into Greek, rank with the best, why should they be despised when purely English? In our opinion ladies-slipper is in no respect inferior to cypripedium; nor would orchestridia be better than opera-girls.

In Dr. Roxburgh's essay on the scitamenia, this plant is referred to the genus globba, with which it is certainly a near affinity, but in our opinion, Dr. Sims's reasons for separating it are quite sufficient.

Cluytia *alaternoides.* A plant of no great beauty, but no intelligible representation of it was before extant. This name was originally *clutia*, and was given by Boerhaave, in honor of a Dutch professor, Cluyt; and very properly changed by Mr. Dryander to *cluytia*, which, while it agrees better with the botanist's name prevents its being confounded with *clusia.*

Lobelia *gigantea.* This has been supposed to be the *tupa* of Feuille, one of the most poisonous plants upon record; smelling to the flowers, proving, according to the Holy Father, violently emetic; and rubbing the eyes with the fingers, accidentally smeared with the juice, infallibly destroying the eyes. Dr. Sims, indeed, found no inconvenience from dissecting, as well as smelling to the flowers of this plant; which, however, he has given good reasons for supposing not the same species as the one described, and figured by Father Deuillee.

Stapelia *geminata.* This has been before figured by Masson, but Mr. Edward's drawings are so superior, that we cannot call them superfluous.

Potentilla *clusina.* The petals are not so round in this as in Jacquin's figure, and are abcordal, in which respect Clusius's own figure corresponds.

Menyanthes *sarmentosa.* A water plant from New South Wales.

Panax *quinquefolia.* The celebrated Ginseng of the Chinese; so famed through China and Japan for its medicinal virtues, particularly as a restorative; and so totally neglected by the medical practitioners of Europe, though easily attainable from North America.

Panax *pusilla.* This is a much smaller species than the last, and has a round root, very like a small potatoe.

Fumaria *formosa.* This is a third plant occurring in this report, and another still remains, which was introduced from the north-west coast of America, by Mr. Archibald Menzies, and a very valuable addition to our gardens it seems to be; being easily propagated perfectly hardy, and very beautiful, both in foliage and flower.

