# Errata

**Published:** 1810-12

**Authors:** 


ERRATA.—Page 529, *for* vivalve, *read* bivalve.

———, — Goodio, ——— Goodia.

——— 530, — fermosa, ——— formosa.

